# Supplementary material for: Iterative improvement in the automatic modular design of robot swarms
Source: PeerJ Comput Sci. 2020 Dec 7;6:e322. doi: 10.7717/peerj-cs.322 (PMC7924708; doi:10.7717/peerj-cs.322)
Supplement: Supplemental Information 3 [file peerj-cs-06-322-s003.zip › argos3/doc/api/standalone/a00501_source.html]

ARGoS: plugins/robots/generic/control\_interface/ci\_leds\_actuator.h Source File


- Main Page
- Related Pages
- Namespaces
- Classes
- Files

- File List
- File Members

# plugins/robots/generic/control\_interface/ci\_leds\_actuator.h

Go to the documentation of this file.

```
00001 
00007 #ifndef CCI_LEDS_ACTUATOR_H
00008 #define CCI_LEDS_ACTUATOR_H
00009 
00010 namespace argos {
00011    class CCI_LEDsActuator;
00012 }
00013 
00014 #include <argos3/core/control_interface/ci_actuator.h>
00015 #include <argos3/core/utility/datatypes/color.h>
00016 
00017 namespace argos {
00018 
00019    class CCI_LEDsActuator : public CCI_Actuator {
00020 
00021    public:
00022 
00023       typedef std::vector<CColor> TSettings;
00024 
00025    public:
00026 
00027       CCI_LEDsActuator() {}
00028 
00029       virtual ~CCI_LEDsActuator() {}
00030 
00034       size_t GetNumLEDs() const;
00035 
00044       virtual void SetSingleColor(UInt32 un_led_number,
00045                                   const CColor& c_color);
00046       
00055       virtual void SetAllColors(const CColor& c_color);
00056 
00067       virtual void SetAllColors(const TSettings& c_colors);
00068 
00076       virtual void SetSingleIntensity(UInt32 un_led_number,
00077                                       UInt8 un_intensity);
00078 
00085       virtual void SetAllIntensities(UInt8 un_intensity);
00086 
00087 #ifdef ARGOS_WITH_LUA
00088       virtual void CreateLuaState(lua_State* pt_lua_state);
00089 #endif
00090 
00091    protected:
00092 
00093       TSettings m_tSettings;
00094 
00095    };
00096 
00097 }
00098 
00099 #endif
```

---

Generated on 10 Jul 2018 for ARGoS by 
 1.6.1
